# Evaluation of *in vitro* activity of ceftolozane/tazobactam and comparators against recent clinical bacterial isolates, and genomics of *Pseudomonas aeruginosa, Klebsiella pneumoniae* and *Escherichia coli* isolates that demonstrated resistance to ceftolozane/tazobactam: data from Kuwait and Oman

**DOI:** 10.1093/jacamr/dlac035

**Published:** 2022-04-21

**Authors:** Wadha Alfouzan, Rita Dhar, Jalila Mohsin, Feryal Khamis, Eiman Mokaddas, Abrar Abdullah, Abu Salim Mustafa, Aurelio Otero, Paulette Wanis, Samar Hussien Matar, Sherif Khalil, Irina Alekseeva, Katherine Young

**Affiliations:** 1 Microbiology Unit, Department of Laboratories, Farwania Hospital, Kuwait City, Kuwait; 2 Department of Microbiology, Faculty of Medicine, Kuwait University, Kuwait City, Kuwait; 3 The Royal Hospital, Masqat, Oman; 4 Microbiology Unit, Department of Laboratories, Ibn Sina Hospital, Kuwait City, Kuwait; 5 Microbiology Unit, Department of Laboratories, Amiri hospital, Kuwait City, Kuwait; 6 Merck & Co. Inc., Kenilworth, NJ 07033, USA; 7 Merck Sharp & Dohme IDEA Middle East, Dubai Healthcare City, AlFaris building #39, 4th floor, MSD Office, Dubai, UAE

## Abstract

**Background:**

The treatment options for infections caused by MDR Gram-negative bacteria have been limited, especially for infections caused by bacteria that produce carbapenemases and/or ESBLs. Ceftolozane/tazobactam is a cephalosporin/β-lactamase inhibitor developed to treat Gram-negative bacteria.

**Methods:**

Ceftolozane/tazobactam and 14 comparators (amikacin, aztreonam, cefepime, cefotaxime, cefoxitin, ceftazidime, ceftriaxone, ciprofloxacin, colistin, ertapenem, imipenem, levofloxacin, meropenem and piperacillin/tazobactam) were evaluated against *Pseudomonas aeruginosa* and Enterobacterales isolates collected from Kuwait and Oman (*n *= 606) during 2016–17. In addition, further analysis of resistance mechanisms to ceftolozane/tazobactam was done utilizing WGS. Non-susceptible isolates from ceftolozane/tazobactam surveillance were selected for analysis. Overall, 35 strains underwent WGS.

**Results:**

Among isolates from Kuwait, susceptibility of *P. aeruginosa*, *Escherichia coli* and *Klebsiella pneumoniae* to ceftolozane/tazobactam was 79.8%, 95.7% and 87.5%, respectively, and from Oman was 92.3%, 93.1% and 88.5%, respectively. No *P. aeruginosa* with a ceftolozane/tazobactam MIC <32 mg/L encoded β-lactamases besides normal chromosomal enzymes (PDC variants or OXA-50-like) whereas all but one *P. aeruginosa* isolate with MIC >32 mg/L encoded either MBLs (60%), VEB-1 (19%) or additional OXAs (3.7%).

**Conclusions:**

Colistin followed by ceftolozane/tazobactam showed the greatest activity against *P. aeruginosa*. Enterobacterales showed more susceptibility to ceftolozane/tazobactam than to piperacillin/tazobactam, but meropenem and colistin showed better activity.

## Introduction

The worldwide increase of MDR Gram-negative bacteria (GNB) is causing a global concern and an economic burden.^1–4^ It is, therefore, important to understand the epidemiology of this multifaceted problem in order to prevent the spread of MDR GNB. As treatment choices for infections caused by these organisms remain limited, patients will continue to present with difficult-to-treat infections caused by MDR GNB. Serious infections, including bloodstream infections, hospital-acquired pneumonia (HAP), ventilator-associated pneumonia (VAP), complicated urinary-tract infections (cUTIs), and complicated intra-abdominal infections (cIAIs) are commonly caused by MDR GNB, often resulting in high morbidity and mortality. Among these organisms, Enterobacterales (especially *Escherichia coli* and *Klebsiella pneumoniae*) and *Pseudomonas aeruginosa* have emerged as major causes of nosocomial infections.^5–7^ Newer antibiotic drugs with anti-GNB activity are being developed, albeit at a slower pace, to combat such infections and among them ceftolozane/tazobactam was recently approved for treatment of cIAIs, cUTIs including acute pyelonephritis, HAP and VAP by the US FDA.^8–10^ and EMA. Ceftolozane/tazobactam is a cephalosporin/β-lactamase inhibitor developed for use against infections caused by GNB, including MDR *P. aeruginosa* isolates that harbour chromosomally encoded resistance mechanisms such as *Pseudomonas*-derived cephalosporinase (PDC), porin defects and upregulated efflux transport systems and ESBL-producing Enterobacterales. The chemical structure of ceftolozane, though similar to that of ceftazidime, possesses a modified side-chain at the 3-position of the cephem nucleus that confers potent antipseudomonal activity. Ceftolozane/tazobactam displays reduced activity against *P. aeruginosa* isolates carrying carbapenemases and against Enterobacterales carrying AmpC β-lactamases, serine carbapenemases, metallo-β-lactamases and OXA-type carbapenemases.^11,12^ Therapy should be individualized based on genotypes of resistance, susceptibility profiles, disease severity and patient characteristics. More studies are needed to guide effective treatment for infections caused by MDR GNB. The purpose of this work was to evaluate the antibacterial activity of ceftolozane/tazobactam against *P. aeruginosa* (*n* = 150) and Enterobacterales isolates (*n* = 456) ceftolozane/tazobactam-resistant isolates utilizing a WGS approach.

## Materials and methods

### Study design

GNB isolates from the EM200 study collected by International Health Management Associates Inc. (IHMA) from 2016 to 2017 were selected for analysis. The sites each collected up to 250 consecutive Gram-negative pathogens from patients with lower respiratory, intra-abdominal, urinary tract, bloodstream and other infections. The study evaluated the *in vitro* activity of ceftolozane/tazobactam and 14 comparator compounds against GNB isolates (*P. aeruginosa, K. pneumoniae* and *E. coli*) collected from 10 countries: in Europe (Turkey, Ukraine), Middle East (Israel, Jordan, Kuwait, Oman, Saudi Arabia, United Arab Emirates) and Africa (Morocco, South Africa). For selected ceftolozane/tazobactam non-susceptible strains from Kuwait and Oman, demographics, susceptibilities and WGS data were retrieved from the IHMA database. We evaluated the antibacterial activity of ceftolozane/tazobactam and 14 comparator compounds against a collection of these isolates from Kuwait and Oman (*n *= 606). The secondary purpose of this work was to investigate mechanisms of resistance to ceftolozane/tazobactam utilizing a WGS approach. In Kuwait and Oman 27 *P. aeruginosa* isolates (25 from Kuwait and 2 from Oman) and 8 Enterobacterales isolates (6 from Kuwait and 2 from Oman) underwent WGS.

### Antimicrobial susceptibility testing

MICs of ceftolozane/tazobactam, amikacin, aztreonam, cefepime, cefotaxime, cefoxitin, ceftazidime, ceftriaxone, ciprofloxacin, colistin, ertapenem, imipenem, levofloxacin, meropenem and piperacillin/tazobactam were determined by broth microdilution following the CLSI reference method.^13,14^ Interpretive criteria followed CLSI 2020 guidelines^14^ for all compounds except colistin for which the EUCAST 2017 breakpoint was used for Enterobacterales.^15^ Quality control (QC) testing was performed each day of testing as specified by CLSI using *E. coli* ATCC 25922, *P. aeruginosa* ATCC-27853 and *K. pneumoniae* ATCC-700603. All QC data were within CLSI approved ranges.^14^

### DNA extraction and WGS analysis

Isolates were cultured overnight at 37°C on tryptic soy agar with 5% sheep blood. A single colony from each plate was inoculated into 5 mL of brain heart infusion broth and incubated at 37°C with 200 rpm shaking overnight. A broth sterility control was incubated concurrently. Genomic DNA was purified using DNeasy UltraClean kits. Extracted DNA was sent to an external sequencing provider for library preparation using Nextera kits and sequencing using a HiSeq sequencing instrument with 2 × 150 bp pair-end reads with a target coverage depth of approximately 150×. All analyses were carried out using Qiagen’s CLCBio Genomics Workbench version 11.

### Analysis of β-lactamase variants

For β-lactam resistance gene identification, *de novo* assemblies of each genome were queried. To detect better highly diverse *ampC* genes for which there are few variants defined, the threshold for minimum nucleotide sequence identity and minimum sequence length were set to 72% and 80%, respectively. However, results that were less than 100% identical or did not contain the full-length sequence were appended as such for clarity. Only β-lactam resistance genes were identified this way. Nucleotide sequences for all PDC variants assigned in GenBank were collected from NCBI (BioProject 313047) and added to the database used to identify resistance genes. *K. pneumoniae* is known to frequently encode a chromosomal copy of *bla*_SHV_. Due to the difficulties associated with differentiating multiple copies of the same gene by Illumina WGS, all reads from *K. pneumoniae* isolates were aligned directly to the *bla*_SHV-1_ gene and the sites associated with an ESBL phenotype (G238S and E240K by Ambler numbering) were reviewed manually to ensure the presence of a chromosomally encoded SHV did not mask the presence of a horizontally transferred (plasmid-encoded) ESBL copy of SHV.

### Analysis of porins

For porin gene identification, *ompC* and *ompF* in *E. coli*, *ompK35* and *ompK36* in *K. pneumoniae* and *oprD* in *P. aeruginosa* were searched by tblastn in the *de novo* assemblies of the genomes. The minimum threshold for E-values was 10E-75, however only the hit with the lowest E-value in each genome was assessed. Lesions were defined as changes in the coding sequence of a gene that would result in a premature stop codon. For PBP gene analysis, reference sequences for the protein products of *ftsI* were searched on a species-specific basis in *de novo* assemblies of each genome. In brief, tblastn was used to find the gene with the lowest E value to the reference sequence, for which mutations encoding amino acid changes were identified.

### Molecular typing: MLST

For MLST, the best matching complete prokaryotic genome from GenBank was identified computationally for each set of genomic reads and used for guided assembly. The appropriate MLST scheme and allelic profile of each of the guided assemblies was determined. A minimum coverage depth of 30× for each of the seven loci was exceeded in every genome. Due to the large number of sequence types identified, phylogenetic trees were used to supplement MLST.

### Ethical approval

Ethical approval was not required.

## Results

### Antimicrobial susceptibility testing

A total of 510 and 96 clinical GNB isolates from Kuwait and Oman, respectively, were tested for antimicrobial activity against a set of 15 antimicrobial agents including ceftolozane/tazobactam, amikacin, aztreonam, cefepime, cefotaxime, cefoxitin, ceftazidime, ceftriaxone, ciprofloxacin, colistin, ertapenem, imipenem, levofloxacin, meropenem and piperacillin/tazobactam. The results for Kuwait and Oman strains are presented in Tables 1 and 2.

**Table 1.** *In vitro* activity of ceftolozane/tazobactam and comparators against 124 and 26 *P. aeruginosa* isolates from Kuwait and Oman respectively

Antimicrobial agent	Antimicrobial susceptibility (%)^a^			MIC (mg/L)		
	susceptible	intermediate	resistant	MIC_50_	MIC_90_	range
Ceftolozane/tazobactam						
Oman	92.3	0	7.7	1	4	0.5 to >32
Kuwait	79.8	2.4	17.7	1	>32	0.25 to >32
Amikacin						
Oman	92.3	0	7.7	≤4	16	≤4 to >32
Kuwait	78.2	3.2	18.6	≤4	>32	≤4 to >32
Aztreonam						
Oman	61.5	19.2	19.2	8	>16	≤1 to >16
Kuwait	58.1	12.9	29	8	>16	≤1 to >16
Cefepime						
Oman	80.8	3.9	15.4	4	>32	2 to >32
Kuwait	66.1	9.7	24.2	4	>32	≤1 to >32
Ceftazidime						
Oman	80.8	0	19.2	4	>32	≤1 to >32
Kuwait	73.4	1.6	25	4	>32	≤1 to >32
Ciprofloxacin						
Oman	84.6	3.9	11.5	≤0.25	>2	≤0.25 to >2
Kuwait	59.7	4.8	35.5	0.5	>2	≤0.25 to >2
Colistin						
Oman	100	—	0	≤1	≤1	≤1 to ≤1
Kuwait	96.8	—	3.2	≤1	2	≤1 to >4
Imipenem						
Oman	80.8	0	19.2	1	16	≤0.5 to 32
Kuwait	55.7	8.1	36.3	2	>32	≤0.5 to >32
Levofloxacin						
Oman	80.8	3.9	15.4	≤1	>4	≤1 to >4
Kuwait	56.5	8.1	35.5	2	>4	≤1 to >4
Meropenem						
Oman	76.9	3.9	19.2	0.5	>16	≤0.12 to >16
Kuwait	59.7	4.8	35.5	1	>16	≤0.12 to >16
Piperacillin/tazobactam						
Oman	76.9	3.9	19.2	8	>64	≤2 to >64
Kuwait	63.7	16.1	20.2	8	>64	≤2 to >64

a Stratified by country.

**Table 2.** *In vitro* activity of ceftolozane/tazobactam and comparators against 386 and 70 Enterobacterales isolates from Kuwait and Oman, respectively

Antimicrobial agent	Antimicrobial susceptibility^a^																	
	*E. coli*: Kuwait (*n *= 164), Oman (*n *= 29)						*K. pneumoniae*: Kuwait (*n *= 133), Oman (*n *= 26)						other Enterobacterales: Kuwait (*n *= 89), Oman (*n *= 15)					
	%			mg/L			%			mg/L			%			mg/L		
	S	I	R	MIC_50_	MIC_90_	range	S	I	R	MIC_50_	MIC_90_	range	S	I	R	MIC_50_	MIC_90_	range
Ceftolozane/tazobactam																		
Kuwait	95.7	0.6	3.7	0.25	1	≤0.06 to >32	85.7	0.8	13.5	0.5	32	0.12 to >32	88.8	2.3	9	0.5	4	0.12 to 32
Oman	93.1	0	6.9	0.25	2	0.12 to >32	88.5	3.9	7.7	0.5	4	0.25 to >32	86.7	6.7	6.7	0.5	4	0.25 to 16
Amikacin																		
Kuwait	100	0	0	≤4	8	≤4 to 16	95.5	0	4.5	≤4	8	≤4 to >32	98.9	1.1	0	≤4	8	≤4 to >32
Oman	93.1	3.5	3.5	≤4	8	≤4 to >32	92.3	0	7.7	≤4	≤4	≤4 to >32	100	0	0	≤4	16	≤4 to 16
Aztreonam																		
Kuwait	56.1	6.7	37.2	4	>16	≤1 to >16	53.4	3	43.6	2	>16	≤1 to >16	71.9	2.3	25.8	≤1	>16	≤1 to >16
Oman	55.2	3.5	41.4	2	>16	≤1 to >16	53.9	0	46.2	≤1	>16	≤1 to >16	73.3	6.7	20	≤1	>16	≤1 to >16
Cefepime																		
Kuwait	58.5	11	30.5	≤1	>32	≤1 to >32	55.6	9	35.3	2	>32	≤1 to >32	74.2	5.6	20.2	≤1	>32	≤1 to >32
Oman	55.2	6.9	37.9	≤1	>32	≤1 to >32	53.9	7.7	38.5	≤1	>32	≤1 to >32	73.3	13.3	13.3	≤1	16	≤1 to >32
Cefotaxime																		
Kuwait	50	0.6	49.4	≤1	>32	≤1 to >32	48.9	0.8	50.4	4	>32	≤1 to >32	61.8	3.4	34.8	≤1	>32	≤1 to >32
Oman	44.8	10.3	44.8	2	>32	≤1 to >32	53.9	0	46.2	≤1	>32	≤1 to >32	73.3	0	26.7	≤1	>32	≤1 to >32
Cefoxitin																		
Kuwait	70.7	15.2	14	8	>16	≤2 to >16	71.4	9	19.6	4	>16	≤2 to >16	60.7	0	39.3	4	>16	≤2 to >16
Oman	79.3	6.9	13.8	8	>16	4 to >16	88.5	3.9	7.7	4	16	≤2 to >16	33.3	6.7	60	>16	>16	≤2 to >16
Ceftazidime																		
Kuwait	60.4	12.2	27.4	2	32	≤1 to >32	54.1	5.3	40.6	2	>32	≤1 to >32	71.9	5.6	22.5	≤1	32	≤1 to >32
Oman	62.1	6.9	31	≤1	>32	≤1 to >32	53.9	3.9	42.3	≤1	>32	≤1 to >32	73.3	6.7	20	≤1	>32	≤1 to >32
Ceftriaxone																		
Kuwait	48.8	1.8	49.4	2	>32	≤1 to >32	49.6	2.3	48.1	2	>32	≤1 to >32	62.9	2.3	34.8	≤1	>32	≤1 to >32
Oman	55.2	0	44.8	≤1	>32	≤1 to >32	50	0	50	≤1	>32	≤1 to >32	73.3	0	26.7	≤1	>32	≤1 to >32
Ciprofloxacin																		
Kuwait	41.5	1.2	57.3	>2	>2	≤0.25 to >2	60.2	9	30.8	0.5	>2	≤0.25 to >2	46.1	10.1	43.8	2	>2	≤0.25 to >2
Oman	65.5	3.5	31	0.5	>2	≤0.25 to >2	65.4	11.5	23.1	≤0.25	>2	≤0.25 to >2	80	0	20	≤0.25	>2	≤0.25 to >2
Colistin																		
Kuwait	100	—	0	≤1	≤1	≤1 to 2	94	—	6	≤1	≤1	≤1 to 4	41.6	—	58.4	>4	>4	≤1 to 4
Oman	96.6	—	3.5	≤1	≤1	≤1 to 4	100	—	0	≤1	≤1	≤1 to 2	40	—	60	>4	>4	≤1 to 4
Ertapenem																		
Kuwait	97.6	1.2	1.2	≤0.06	≤0.06	≤0.06 to 4	86.5	0.8	12.8	≤0.06	2	≤0.06 to >4	92.1	2.3	5.6	≤0.06	0.5	≤0.06 to >4
Oman	100	0	0	≤0.06	≤0.06	≤0.06 to 0.25	96.2	0	3.9	≤0.06	0.12	≤0.06 to >4	93.3	6.7	0	≤0.06	≤0.06	≤0.06 to 1
Imipenem																		
Kuwait	100	0	0	≤0.5	≤0.5	≤0.5 to 1	94	1.5	4.5	≤0.5	1	<0.5 to >32	68.5	30.3	1.1	1	2	≤0.5 to 4
Oman	100	0	0	≤0.5	≤0.5	≤0.5 to <0.5	96.2	0	3.9	≤0.5	≤0.5	≤0.5 to 4	86.7	6.7	6.7	≤0.5	2	≤0.5 to 4
Levofloxacin																		
Kuwait	42.1	3.1	54.9	>4	>4	≤1 to >4	74.4	4.5	21.1	≤1	>4	≤1 to >4	60.7	11.2	28.1	2	>4	≤1 to >4
Oman	65.5	3.5	31	≤1	>4	≤1 to >4	80.8	7.1	11.5	≤1	>4	≤1 to >4	80	6.7	13.3	≤1	>4	≤1 to >4
Meropenem																		
Kuwait	100	0	0	≤0.12	≤0.12	≤0.12 to 0.5	94	0.8	5.3	<0.12	0.5	≤0.12 to >16	97.8	1.1	1.1	≤0.12	≤0.12	≤0.12 to 16
Oman	100	0	0	≤0.12	≤0.12	≤0.12 to ≤0.12	96.2	0	3.9	≤0.12	≤0.12	≤0.12 to >16	100	0	0	≤0.12	≤0.12	≤0.12 to ≤0.12
Piperacillin/tazobactam																		
Kuwait	90.9	4.3	4.9	≤2	16	≤2 to >64	75.9	8.3	15.8	4	>64	≤2 to >64	80.9	10.1	9	≤2	64	≤2 to >64
Oman	86.2	6.9	6.9	≤2	64	≤2 to >64	76.9	3.9	19.2	4	>64	≤2 to >64	93.3	0	6.7	≤2	8	≤2 to >64

a Stratified by country.

In Kuwait ceftolozane/tazobactam inhibited 79.8% of 124 *P. aeruginosa* isolates at the CLSI susceptible breakpoint of ≤4 mg/L, while 59.7% of isolates were susceptible to meropenem, 78.2% to amikacin and 68.7% to piperacillin/tazobactam. Colistin was the most active compound tested, with a 96.8% susceptibility rate (Table 1). Ceftolozane/tazobactam inhibited 95.7% of 164 *E. coli* isolates at the CLSI Enterobacterales susceptibility breakpoint of ≤2 mg/L, while 100% of *E. coli* isolates were susceptible to meropenem, amikacin and colistin, and 90.9% were susceptible to piperacillin/tazobactam (Table 2). Ceftolozane/tazobactam inhibited 85.7% of 133 *K. pneumoniae* isolates; 94.0% of *K. pneumoniae* isolates were susceptible to meropenem, 95.5% to amikacin, 94.0% to colistin and 75.9% to piperacillin/tazobactam (Table 2). Ceftolozane/tazobactam inhibited 88.8% of 89 other Enterobacterales; 97.8% of these isolates were susceptible to meropenem, 98.9% to amikacin and 80.9% to piperacillin/tazobactam (Table 2). Among Oman isolates, ceftolozane/tazobactam inhibited 92.3% of 26 *P. aeruginosa* isolates at the CLSI susceptibility breakpoint of ≤4 mg/L; 76.9% of these isolates were susceptible to meropenem, 92.3% to amikacin and 76.9% to piperacillin/tazobactam. Colistin was the most active compound tested, with 100% isolates susceptible (Table 1). Among Oman isolates, ceftolozane/tazobactam inhibited 93.1% of 20 *E. coli* isolates at the CLSI Enterobacterales susceptibility breakpoint of ≤2 mg/L; 100% of *E. coli* isolates were susceptible to meropenem, 93.1% to amikacin, 96.9% to colistin and 86.2% to piperacillin/tazobactam. Ceftolozane/tazobactam inhibited 88.5% of 26 *K. pneumoniae* isolates compared with 96.2% of isolates susceptible to meropenem, 92.3% to amikacin, 100% to colistin and 76.9% to piperacillin/tazobactam. Ceftolozane/tazobactam inhibited 86.7% of 15 other Enterobacterales isolates. All these isolates were susceptible to meropenem and amikacin, and 93.3% of them were susceptible to piperacillin/tazobactam (Table 2).

### Organism selection and demographics

Among the 510 GNB isolates from Kuwait, 31 strains (6%) tested non-susceptible, and among the 96 isolates from Oman, 4 strains (4.1%) tested non-susceptible (MIC values ≥8 mg/L) to ceftolozane/tazobactam by antimicrobial susceptibility testing (Table 3). These isolates were selected for further analysis. The various sources of these isolates (number/percentage of total) from Kuwait were: respiratory (13/41.9%), UTI (11/35.5%), SSTI (3/9.7%), IAI (2/6.5%) and body aspirate (cerebrospinal fluid, abscess aspirates from various anatomical locations, pleural fluid, ascites, bone biopsy, tissue biopsy from sterile anatomical sites such as brain, liver, spleen, and lymph nodes, bone marrow aspirate, synovial biopsy and synovial fluid samples) (2/6.5%) whereas of four strains from Oman two were isolated from intra-abdominal samples, one from urine and one from respiratory samples. The demographic information for the isolates that were selected for WGS is as follows:

- For *P. aeruginosa* isolates (*n *= 25) from Kuwait, the age of patients ranged from 6 to 87 years, 11 patients were male while 14 were female and culture-positive samples were 9 from urine, 11 from lower respiratory tract, 3 from wound cultures and 2 from pus samples.
- For two *P. aeruginosa* isolates from Oman, the age of patients ranged from 18 to 34 years and positive cultures were one isolate from respiratory origin and the other from intra-abdominal source.

**Table 3.** Ceftolozane/tazobactam MIC distribution for *P. aeruginosa* and Enterobacterales isolates included in WGS, stratified by country

Isolate and country of origin	No. of isolates	Ceftolozane/tazobactam MIC (mg/L)			
		8	16	32	>32
*P. aeruginosa*					
Kuwait	25	3	1	—	21
Oman	2	—	—	—	2
Enterobacterales					
Kuwait	6	1	1	—	4
Oman	2	—	1	—	1

For *K. pneumoniae,* the demographics for the isolates that underwent WGS were as follows:

- Three isolates from Kuwait were selected, for which the age of patients ranged from 52 to 62 years, one isolate was from a male and two from females and the positive cultures were obtained from urine, respiratory and intra-abdominal samples.
- For two isolates from Oman, the age of patients ranged from 58 to 72 years, one isolate was from a male and the other from a female and the positive cultures were obtained from urine and intra-abdominal samples.

For *E. coli* the demographics for the isolates that underwent WGS were as follows:

- For three isolates from Kuwait that were selected, the age of patients ranged from 20 to 72 years, one isolate was from a male and two were from females and the positive cultures were obtained from urine, respiratory and intra-abdominal samples.
- No *E. coli* isolate from Oman underwent WGS.

### WGS

Overall, 35 isolates underwent WGS and analysis. The genomes of 27 *P. aeruginosa* strains (25 isolates from Kuwait and 2 isolates from Oman) (Table 4) that were completely sequenced showed the presence of *bla*_VIM-2_ in 15 (55.6%), *bla*_OXA-4_ in 10 (37%), *bla*_OXA-10_ in 6 (22%), *bla*_VEB-1_ in 5 (18.5%), *bla*_LCR-1_ in 4 (14.8%), *bla*_OXA-2_ in 3 (11.1%) and *bla*_VIM-6_ in 1 (3.7%). The *bla*_OXA-50-like_ gene was found in 24 (88.9%) of the strains; however, this chromosomally encoded class D enzyme is not associated with β-lactam resistance in *P. aeruginosa*.^16^ Most *P. aeruginosa* isolates presented a combination of genes, e.g. *bla*_VIM-2_ + *bla*_OXA_ + *bla*_PDC_. Independently, class D β-lactamases were found in 14.8% of ceftolozane/tazobactam non-susceptible *P. aeruginosa* isolates (Table 4). Sequencing of the *bla*_PDC_ gene revealed the occurrence of 10 PDC variants (8 from Kuwait and 2 from Oman), different PDC variants with PDC-119-like (40%) and PDC-252-like (40%) being produced by most of the ceftolozane/tazobactam non-susceptible strains from Kuwait. No PDC allele observed contains amino acid substitutions previously identified to be associated with ceftolozane/tazobactam resistance. The *oprD* gene sequence revealed mutations in 17 (68%) of 25 non-susceptible *Pseudomonas* isolates from Kuwait. There was no relationship between the disruption of the *oprD* gene and ceftolozane/tazobactam MIC, since isolates with lower MICs also had lesions in *oprD*. Genotyping by MLST showed that these *P. aeruginosa* strains from Kuwait belonged to ST233 (44%), followed by ST357 (20%) and ST2613 (16%), and all others (20%) were detected as single isolates and included ST272, ST244, ST499, ST3582 and ST671 while the two strains from Oman belonged to ST664 and ST207, respectively.

**Table 4.** WGS data for P. aeruginosa isolates from Kuwait and Oman

Isolates stratified by country	IHMA number	C/T MIC (mg/L)	OprD	PBP3 (*ftsI*)^a^	WGS lactamase summary (≥72% identity; ≥80% CDS)^b^	Class C (intrinsic)	MLST^c^
Kuwait							
1	1562186	8	Frameshift, 195 AA protein	WT	PDC-109	PDC-109	499
2	1562187	>32	No lesion	WT	PDC-119-like; OXA-4; VIM-2	PDC-119-like	233
3	1562188	>32	No lesion	WT	PDC-252-like; OXA-4; VIM-2	PDC-252-like	233
4	1562192	>32	Q158STOP	WT	PDC-119-like; OXA-4; VIM-2	PDC-119-like	233
5	1562196	>32	Frameshift, 359 AA protein	A539T	PDC-252-like; VIM-2; OXA-4	PDC-252-like	233
6	1562198	>32	No lesion	WT	PDC-35; LCR-1; VIM-2; OXA-2	PDC-35	2613
7	1572968	>32	No lesion	WT	PDC-252-like; VIM-2	PDC-252-like	3482
8	1572981	>32	No lesion	WT	PDC-119-like; VIM-2;	PDC-119-like	233
9	1572982	>32	W138STOP	WT	PDC-119-like; OXA-4-like; VIM-2	PDC-119-like	233
10	1572988	>32	Frameshift, 350 AA protein	WT	PDC-35; OXA-2; VIM-6; OXA-10	PDC-35	2613
11	1607795	8	No lesion	WT	PDC-30	PDC-30	671
12	1607809	>32	Disrupted by insertion	WT	PDC-119-like; OXA-4; VIM-2	PDC-119-like	233
13	1607996	>32	No lesion	WT	PDC-35; LCR-1; VIM-2; OXA-2	PDC-35	2613
14	1652654	>32	Frameshift, 354 AA protein	WT	PDC-252-like; OXA-4; VIM-2	PDC-252-like	233
15	1652655	>32	Disrupted by insertion	WT	PDC-252-like; OXA-4; VIM-2	PDC-252-like	233
16	1652663	>32	Frameshift, 135 AA protein	WT	PDC-11; OXA-10; VEB-1	PDC-11	357
17	1723966	>32	Frameshift, 135 AA protein	WT	PDC-11; OXA-10; VEB-1	PDC-11	357
18	1724010	>32	Frameshift, 135 AA protein	WT	PDC-11; VEB-1; OXA-10	PDC-11	357
19	1724024	>32	No lesion	WT	PDC-35; OXA-2; VIM-2; LCR-1	PDC-35	2613
20	1724321	>32	Frameshift, 135 AA protein	WT	PDC-11; OXA-10; VEB-1	PDC-11	357
21	1724327	>32	Frameshift, 135 AA protein	WT	PDC-11; OXA-10; VEB-1	PDC-11	357
22	1734172	8	E176STOP	WT	PDC-212-like	PDC-212-like	272
23	1734205	>32	Disrupted by insertion	WT	PDC-252-like; OXA-4; VIM-2	PDC-252-like	233
24	1734215	>32	Frameshift, 354 AA protein	WT	PDC-119-like; OXA-4; VIM-2	PDC-119-like	233
25	1734223	16	Q424STOP	WT	PDC-279-like	PDC-279-like	244
Oman							
1	1688133	>32	Frameshift, 218 AA protein	L346M; F533L	PDC-98; OXA-10; OXA-1-like	PDC-98	664
2	1688134	>32	No lesion	WT	PDC-30	PDC-30	207

AA, amino acid; C/T, ceftolozane/tazobactam.

a Amino acid changes are greater relative to reference sequences.

b Threshold for β-lactamase gene inclusion was 72% and 80% for minimum nucleotide sequence identity and minimum sequence length, respectively.

c Novel MLSTs were given sequential designations for clarity.

WGS of *K. pneumoniae* strains with MIC ≥8 mg/L identified five unique STs among the three (ST831, ST336 and ST985) *K. pneumoniae* isolates from Kuwait and two (ST1658, ST231) *K. pneumoniae* isolates from Oman. Though each ST was only seen in one isolate, all five isolates carried the ESBL gene *bla*_CTX-M-15_, which was present in combination with *bla*_TEM-1_ (*n *= 2) and *bla*_SHV_ genes (*n *= 5). Among carbapenemase genes, while no *bla*_KPC_ genes were found, *bla*_NDM-1_ and *bla*_OXA-48_ were detected in one isolate each. The porin gene profiles of these five isolates showed that while mutations in the *ompF*-like porin (OmpK35) were detected in both strains from Oman, mutation in the *ompC*-like porin (OmpK36) was detected in only one *K. pneumoniae* isolate from Kuwait (Table 5).

**Table 5.** WGS data for *K. pneumoniae* and *E. coli* isolates from Kuwait and Oman

Isolates stratified by country	IHMA number	C/T MIC (mg/L)	OmpC-like	OmpF-like	PBP3(*ftsI*)^a^	β-Lactamase summary (72% identity; 80% coverage)^b^	MLST
Kuwait							
1. *K. pneumoniae*	1562167	16	No lesion	No lesion	WT	CTX-M-15; OXA-1; OXA-48; SHV-1-like; TEM-1B	831
2. *K. pneumoniae*	1572948	>32	No lesion	No lesion	WT	CTX-M-15; NDM-1; OXA-9-like; SHV-11; TEM-1B	336
3. *K. pneumoniae*	1724041	>32	Frameshift, 70 AA protein	No lesion	WT	CTX-M-15; OXA-1; SHV-83; TEM-1B-like	985
Oman							
1. *K. pneumoniae*	1688174	16	No lesion	Frameshift, 212 AA protein	WT	CTX-M-15; OXA-1; SHV-1; TEM-1B	1658
2. *K. pneumoniae*	1688181	>32	No lesion	Frameshift, 107 AA protein	WT	CTX-M-15; OXA-232; SHV-ESBL; TEM-1B	231
Kuwait							
1. *E. coli*	1572932	>32	No lesion	No lesion	E149D; T233A; V332I; 3331NS-YR1K; A413V	CMY-7-like; EC-like; TEM-34-like	7395
2. *E. coli*	1723961	8	No lesion	No lesion	E149D; Q227H; T233A; V332I; 3331NS- YR1N; E349K; 1532L	CTX-M-15; EC-like; OXA-1-like; TEM-1B	361
3. *E. coli*	1734194	>32	No lesion	No lesion	E149D	CTX-M-15; EC-like; TEM-1B	131

AA, amino acid; C/T, ceftolozane/tazobactam.

a Amino acid changes are greater relative to reference sequences.

b Threshold for β-lactamase gene inclusion was 72% and 80% for minimum nucleotide sequence identity and minimum sequence length, respectively.

c Novel MLSTs were given sequential designations for clarity.

Among three *E. coli* isolates from Kuwait that were examined by WGS, three unique STs (ST7395, ST361 and ST131) were identified. Horizontally transferred β-lactamases were identified in three of three (100%) isolates with ceftolozane/tazobactam MIC values ≥8 mg/L. All these strains carried genes for one or more of the following β-lactamases: EC-like (Class C β-lactamase intrinsic to *E. coli*), CTX-M, CMY-7-like, OXA-1-like, TEM-1B and TEM-34-like. Four amino acid insertions (‘YRIN’ or ‘YRIK’) at position 333 of PBP3 were identified in two of three (66.6%) isolates. No mutations were observed in the gene encoding OmpC or OmpF in any of the *E. coli* strains (Table 5).

## Discussion

The dissemination of MDR GNB and XDR GNB strains compromises selection of appropriate antimicrobial treatments resulting in significant morbidity and mortality.^17–19^ It has been shown that these pathogens develop resistance to most available antibiotics by selection of mutations on chromosomal genes and from the increasing prevalence of transferable resistant determinants, especially those encoding class B carbapenemases (metallo-β-lactamases) or ESBLs, frequently co-transferred with genes encoding resistance to other antibiotics.^20^ The recent introduction of ceftolozane/tazobactam, which is stable against *P. aeruginosa* AmpC hydrolysis (PDC), comes as a respite to the dilemma of selecting an effective antibiotic against MDR/XDR GNB, including carbapenem resistant *P. aeruginosa* (not producing carbapenemases).

The *K. pneumoniae* isolates from Kuwait and Oman exhibited susceptibility to ceftolozane/tazobactam of 85.7% and 88.5%, respectively, with MICs ranging from 0.12 to >32 mg/L. *E. coli* isolates from Kuwait and Oman in our study demonstrated similar susceptibility rates to ceftolozane/tazobactam (95.7% and 93.1%, respectively, with MIC range of ≤0.06 to >32 mg/L). Concerning data from the region, a study from Lebanon showed similar ceftolozane/tazobactam activity against ESBL-producing *E. coli* and *K. pneumoniae* strains (MIC_90_ = 1–1.5 mg/L) with susceptibility rates of 100% and 96%, respectively.^21^ In a recent study from Qatar, an overall susceptibility of *P. aeruginosa* isolates against ceftolozane/tazobactam was found to be 62.9% whereas only <50% of XDR strains were found to be susceptible to ceftolozane/tazobactam.^22^ In contrast, in a large study from the USA, ceftolozane/tazobactam was found to be one of the most active agents against *P. aeruginosa*, retaining activity against MDR and XDR strains with susceptibility rates varying from 95.1% to 98.2%.^23^ In comparison, lower activity of ceftolozane/tazobactam was reported for ESBL-producing Enterobacterales, with a susceptibility range of 85%–93.3%.^24^ However, while ceftolozane/tazobactam shows potent *in vitro* activity against *Pseudomonas* spp. and Enterobacterales, baseline genes encoding resistance including *bla*_MBL_s, *bla*_VEB_ and additional *bla*_OXA_ genes were detectable. The emergence of resistance to ceftolozane/tazobactam and some other newer antibiotics is of particular concern and needs to be monitored closely.^25,26^

Bacteria demonstrate diverse mechanisms for developing resistance such as degrading antibiotics, modifying the antibiotic target site or modulating the influx/efflux of antibiotic into or out of the bacterial cell.^27^ WGS is an emerging tool used for advanced molecular epidemiological investigations such as accurate detection and characterization of existing or emergent resistance determinants, especially involving MDR organisms. Adoption of genotyping can help in better understanding the mechanism(s) of resistance among virulent genotypes and elucidation of transmission routes,^28–30^ which forms an essential aspect of public health surveillance to combat the spread of antimicrobial-resistant bacteria.^31^ The molecular epidemiology and resistance mechanisms of ceftolozane/tazobactam-resistant strains of *P. aeruginosa*, *K. pneumoniae* and *E. coli* from Kuwait and Oman were determined by WGS. Diverse STs of *P. aeruginosa* isolates from Kuwait and Oman were observed. Since carbapenemase production is considered to be one of the resistance mechanisms to ceftolozane/tazobactam, resistance to carbapenems is being used as a marker of potential carbapenemase production.^20^ Similar to an earlier study where the VIM-2 type carbapenemase was the most common followed by the IMP type, our study showed that VIM-2 was the most common carbapenemase. However; independently, OXA-type β-lactamases were found in only 4 of 27 non-susceptible *P. aeruginosa* isolates (14.8%). In most cases this enzyme was associated with other carbapenemases and ESBLs. Recent studies have revealed that besides PDC overexpression, β-lactam resistance development may result from mutations leading to the structural modification of PDC or other β-lactamases. Previously published studies have identified certain amino acid substitutions, as well as deletions, in the omega-loop region of the intrinsic *P. aeruginosa ampC* gene (also known as *bla*_PDC_) that are associated with decreased susceptibility to ceftolozane/tazobactam.^26,32–35^ PDC analysis revealed that all of the strains exhibited the PDC gene whereas no mutations in the intrinsic *ampC* gene (*bla*_PDC_), which have been reported previously to be associated with increased ceftolozane/tazobactam MICs, were observed. Genetic lesions that introduced a premature stop codon in *oprD* were identified frequently (68%); however, this feature is not associated with resistance to ceftolozane/tazobactam, and we also did not observe any relationship between ceftolozane/tazobactam MIC and lesions in *oprD.*^12^ For the *P. aeruginosa* isolates in which no horizontally transferred β-lactamase was identified (*n *= 4), all from Kuwait, four unique sequence types were determined. This indicates that the elevated ceftolozane/tazobactam MIC values observed among these isolates, which included three isolates with intermediate (8 mg/L) and one with resistant (>8 mg/L) MIC values, is not due to the spread of one highly successful clone. Among those isolates that encoded horizontally transferred β-lactamases, an association was observed between the PDC allele and ST for the PDC-11, PDC-35, PDC-119-like and PDC-252-like alleles. The finding of β-lactamases within the PDC allele groups may indicate clonal spread.

During the past decade there has been a worldwide increase in ESBL-producing and carbapenem-resistant *E. coli*.^36–39^ The mechanisms of resistance to carbapenems include production of carbapenemases, AmpC type enzymes and ESBLs, and membrane impermeability, which can be linked to modification or absence of OmpC or OmpF porin channels.^37,38^ Insertions of four amino acids in PBP3, ‘YRIK/YRIN’, previously reported to decrease susceptibility to select cephalosporins, including ceftazidime.^39^ were identified in 66.6% of our isolates. The other isolate also had mutation in the *ftsI* gene, encoding PBP3. All *E. coli* isolates from Kuwait and Oman in this study were carbapenem susceptible, with rare resistance to ceftolozane/tazobactam. Non-susceptibility of three *E. coli* isolates was attributed to one of several factors including alterations of PBPs due to mutations. In a similar study from Lebanon, the most common type of *E. coli* isolated belonged to ST405, which has been detected in the USA, Japan and Norway.^40^ This strain of *E. coli* has been shown to be associated with the worldwide spread of *bla*_CTX-M-15_ and *acc-(6*′*)-Ib-cr*.^41^ However, among our *E. coli* strains, none belonged to ST405 and while two strains carried genes for CTX-M-15 and one for CMY-7-like cephalosporinase; none of the strains showed any lesion in *ompF*-like and *ompC*-like genes. An *ompC-*like (*ompK36*) gene mutation was identified in one strain from Kuwait and *ompF-*like (*ompK35*) was identified in the two strains from Oman.

### Conclusions

In Kuwait and Oman, ceftolozane/tazobactam showed greater activity against *P. aeruginosa* strains when compared with meropenem and piperacillin/tazobactam. Ceftolozane/tazobactam was more or equally active compared with amikacin. Colistin demonstrated the highest activity against *P. aeruginosa*. Class D carbapenemases were found in all *P. aeruginosa* ceftolozane/tazobactam non-susceptible isolates in combination with MBL or VEB. Mutations in a gene encoding an outer membrane protein (*oprD*) were frequently identified however, no relationship was seen between *oprD* status and ceftolozane/tazobactam MIC.^12^*E. coli* and *K. pneumoniae* showed higher susceptibility to ceftolozane/tazobactam than to piperacillin/tazobactam. It may, therefore, have utility as a carbapenem-sparing antibiotic for the treatment of ESBL-producing Enterobacterales. Meropenem and colistin showed better activity against these isolates from Kuwait and Oman. Resistance of Enterobacterales isolates to ceftolozane/tazobactam could be explained by the presence of β-lactamase combinations, lesions in the OmpC-like and OmpF-like porins and mutation in PBP3-*ftsI.* Reduced susceptibility to ceftolozane/tazobactam in some of the isolates may have been caused by higher levels of β-lactamases, which could have exceeded the available amount of tazobactam.
